# Microbial contamination of toothbrushes during treatment with multibracket appliances

**DOI:** 10.1186/1746-160X-10-43

**Published:** 2014-10-10

**Authors:** Johanna Eichenauer, Julia von Bremen, Sabine Ruf

**Affiliations:** Private practice, Pestalozzistraße 27, 61231 Bad Nauheim, Germany; Department of Orthodontics, Justus-Liebig-University, Schlangenzahl 14, 35392 Giessen, Germany

**Keywords:** Microbial contamination, Toothbrush design, Multibracket therapy

## Abstract

**Introduction:**

It was aimed to assess the retention of caries-associated microorganisms on two different manual toothbrushes (conventional and tapered) and to evaluate the influence of multibracket appliances (MB) on the microbial contamination of the brush head.

**Methods:**

50 MB-patients and 50 subjects without MB received a toothbrush (elmex® interX medium short head or meridol®) plus toothpaste (elmex®) for exclusive use and an information sheet with standardised brushing instructions. After 14 days of regular tooth brushing, the brushes were collected and sluiced in Sputasol solution. The suspension was incubated on selective agar plates and the amount of Streptococcus mutans, lactobacilli and Candida albicans for each brush head was assessed.

**Results:**

Regarding the retention of microorganisms, no differences could be detected between the two bristle designs. However, the amount of S. mutans was significantly higher on brushes used by MB-patients (p < 0.005) than on the brushes of subjects without MB. The number of Lactobacilli and C. albicans was minimal in all cases and below statistical evaluation.

**Conclusions:**

During treatment with MB appliances, toothbrushes were contaminated more intensely with S. mutans independent of bristle design. A more frequent replacement of toothbrushes may thus be recommended for patients undergoing MB-treatment.

## Introduction

As a result of increasing health consciousness and demand for an aesthetic dentition with ideal function the total amount of patients undergoing orthodontic treatment has increased during the last years. According to the guidelines of health care systems over one third of adolescents require orthodontic therapy nowadays [1] and the majority of these treatments is performed with multibracket (MB) appliances. However, particularly treatment with MB appliances is challenging with regard to oral hygiene, which is why white spot lesions remain to be the most frequent undesired side effect of orthodontic treatment. The insertion of fixed appliances alters the oral microbiological profile, thus increasing the risk for caries and gingivitis considerably [2–4]. Furthermore, caries and gingivitis prevention demands greater efforts in toothbrushing, since brushing becomes more complicated as substantial parts of the tooth surface are covered with attachments [5].

An ideal design for toothbrushes used during orthodontic treatment is not yet agreed on [6]. Repeatedly suspected advantages of electrical toothbrushes couldn´t be confirmed [6, 7], so it remains mainly up to the patient what kind of toothbrush he or she prefers and is able to use effectively. For the last couple of years manual toothbrushes with soft conical filaments (tapered bristles) have been available on the market. These are recommended in particular for patients with periodontal problems or after oral surgery. Barnes et al. [8] commented on superior approximal and subgingival cleaning efficacy of brushes with tapered bristles. Furthermore tapered brushes have been shown to be superior to brushes with conventional cylindrical filaments [9, 10]. Thus, it can be assumed that MB-patients using brushes with tapered filaments might reach an improved cleaning efficacy compared to patients using brushes with conventional cylindrical filaments, since areas which are hardly accessible, such as the region between bracket and gingiva, might be reached and cleaned more easily.

Already after the single use of a toothbrush, but even more after the daily usage for weeks or months, the brush head becomes colonised by bacteria of the oral cavity [11–13]. Due to the narrow arrangement of the fine filaments in tapered toothbrushes the retention of moisture and debris is facilitated. In turn, increased microbial contamination of tapered toothbrushes seems likely.

A common risk, especially in adolescent orthodontic patients, is a sucrose-rich nutrition and inadequate oral hygiene, which may lead to a rapid development of decalcifications and an increase of the intraoral amount of *S. mutans* [14]. Since about 50 years, streptococci of the mutans-group have been considered essential bacteria for inducing caries [15], but lactobacilli and *Candida albicans* are also held responsible for the initiation and progress of dental decay [16, 17]. These germs also favour a high-carbohydrate diet and increase in numbers depending on the presence of retentive areas in the mouth [16]. As the development of caries or white spot lesions is common during MB treatment [18], it would be desirable to recommend toothbrushes to orthodontic patients, which retain as little caries-associated microorganisms on the brush heads as possible.

Therefore, it was the aim of the present investigation to evaluate the retention of three caries-associated microorganisms (*S. mutans*, lactobacilli and *Candida albicans*) on two manual toothbrushes differing in their filament design (conventional cylindrical vs. fine tapered) and to assess the influence of a multibracket appliance on the microbial contamination of the brush head.

## Materials and methods

### Two patient samples were analysed

Group MB (multibracket): 50 multibracket (MB)-patients undergoing treatment at the Department of Orthodontics (University of Giessen) with attachments on ≥ 20 teeth (mean age 13.5 years, 14 male, 29 female).

Group nMB (non-multibracket): 50 undergraduate dental students (University of Giessen) without fixed orthodontic appliances (mean age 24.5 years, 12 male, 32 female).

**Figure 1:**
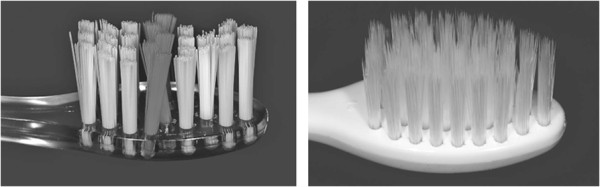
**Design of toothbrush heads of brand-new elmex® (left) and meridol® toothbrushes (right).**

Further inclusion criteria were a healthy or conservatively treated permanent dentition. Subjects with systemic diseases, mental or physical disabilities, caries, periodontic problems or long-term medication were not included. The use of antibiotic or other anti-infective agents was not allowed during the trial. Before beginning, ethical approval was obtained from the ethic committee (University of Giessen, No.140/08). All participants had to give prior written informed consent. If subjects were under 18 years of age, an additional parental consent was obtained. Subsequently subjects were pseudonymised and randomly assigned either a manual toothbrush with tapered filaments (meridol®) or a toothbrush with conventional cylindrical bristles (elmex®). Thus, four groups with 25 participants each were formed: MB-subjects using the elmex® brush (MBe), MB-subjects using the meridol® brush (MBm) and subjects without MB using either the elmex® (nMBe) or meridol® brush (nMBm) (Table 1).Both toothbrushes were multi-tufted, constructed with staple-set tufting and differed in filaments´ shape and diameter. The elmex® toothbrush head was comprised of 27 tufts with nylon filaments of 0.175 and 0.2 mm, which were end-rounded and medium stiff. In contrast the meridol® toothbrush had 37 tufts with conical soft bristles ranging from 0.18 mm at the base to 0.05 mm at the top (GABA, Lörrach, Germany) (Figure 1).

**Table 1:** **Allocation of the experimental study groups**

	elmex ^®^ toothbrush	meridol ^®^ toothbrush
**Participants** ***with***	group **MBe**	group **MBm**
**multibracket appliance**	*(n=25)*	*(n=25)*
**Participants** ***without***	group **nMBe**	group **nMBm**
**multibracket appliance**	*(n=25)*	*(n=25)*

At the start of the trial all subjects received the same toothpaste (elmex®) for exclusive use together with their toothbrush. At the same time point a resealable polythene bag and an instruction sheet were distributed to each participant. Subjects were requested to use the brush twice a day for three minutes, in the morning and evening, for 14 consecutive days. After brushing, the brush head had to be cleaned with running tap water for about 5 seconds. The toothbrush was stored head up until the next brushing sequence. The use of mouthwash was not allowed, whereas the additional use of dental floss or interdental brushes was permitted. Two weeks later the toothbrushes were returned in the sealed polythene bags, and germ isolation was performed immediately. The examiner evaluating the amount of colony-forming units (CFU) was blinded for subjects (group assignment) and toothbrush type.

Together with the used toothbrush the completed questionnaires were returned. Using dichotomous questions (“yes” or “no”), participants were asked whether they had suffered from any illness during the course of the study, if they had taken any medication or if they had used any other toothbrush than the assigned trial brush. If antibiotic or anti-infective medication had been used, this resulted in retrospective exclusion of the study. By means of 100 mm visual analogue scales (VAS), subjective perceptions concerning bleeding tendency, pain and cleaning efficacy were assessed.

The isolation of microorganisms was performed according to the method described by Wetzel et al. [19] and Nies et al. [20]. Brush heads were placed in small glass containers with 10 ml Sputasol solution (Oxoid, Basingstoke, England), sealed with parafilm and sluiced at 25°C, facilitated by ultrasound, for 15 minutes. Following centrifugation of 1 ml at 13000 rpm for 10 minutes, 800 μl supernatant were discarded and the remaining 200 μl were mixed on a vortexer. For selective detection of *S. mutans* and lactobacilli, CRT® bacteria (Ivoclar Vivadent, Schaan, Liechtenstein) was used, which contains a two-side base plate with Mitis Salivarius and Rogosa agars on each of which 20 μl of the solution were plated. Another 20 μl were spread on Sabouraud agar for cultivating *Candida* species. After 72 hours of incubation at 36°C, the colony forming units (CFU) were counted with the aid of an experienced medical laboratory assistant.

### Statistics

The study was designed in collaboration with the Institute for Medical Informatics (University of Giessen). Subjects were allocated to either one of the toothbrushes by using a randomisation list. As a normal distribution of values could not be assumed, non-parametric methods (Wilcoxon- and Median test) were applied. The distribution of the parameters was thus described by means of minimum, maximum, median as well as first and third quartiles. To allow for a better comparability with literature, mean scores and standard deviation were additionally determined. Statistical significance was set at p < 0.05. A possible interaction between toothbrush design and multibracket treatment was assessed using the H-test and logistical regression.

## Results

After 14 days, 87 subjects, 26 male and 61 female, completed the trial according to the inclusion criteria, resulting in a dropout-rate of 13%. The reasons for the dropouts are listed in Table 2. Microbial analyses thus were conducted for 87 brushes. Illness during the brushing interval was stated by 18 subjects, but in these cases a possible use of medication was in accordance with the inclusion criteria. An impact of transient health restrictions on our investigation could not be validated (p = 0.3190). No gender-related difference could be determined with respect to microbial colonisation of the brushes (p = 0.5421). In total the elmex® toothbrush was used by 47 patients (23 MB, 24 nMB), the meridol® brush by 40 participants (20 MB, 20 nMB) (Table 3).

**Table 2:** **Dropout reasons**

Reason	n
termination by subjects	3
irregular use of toothbrush	2
return of brush ≥ 24h late	2
use of antibiotics	5
mould growth on agar plate	1
**Total**	**13**

**Table 3:** **Number and gender of subjects in the four experimental groups**

Gender	Male	Female	Total
	n	n	n
MBe	9	14	23
MBm	5	15	20
nMBe	8	16	24
nMBm	4	16	20
**Total**	**26 (30%)**	**61 (70%)**	**87**

(MBe= MB and elmex® toothbrush, MBm= MB and meridol® toothbrush, nMBe= no MB and elmex® toothbrush, nMBm= no MB and meridol® toothbrush).

**Figure 2:**
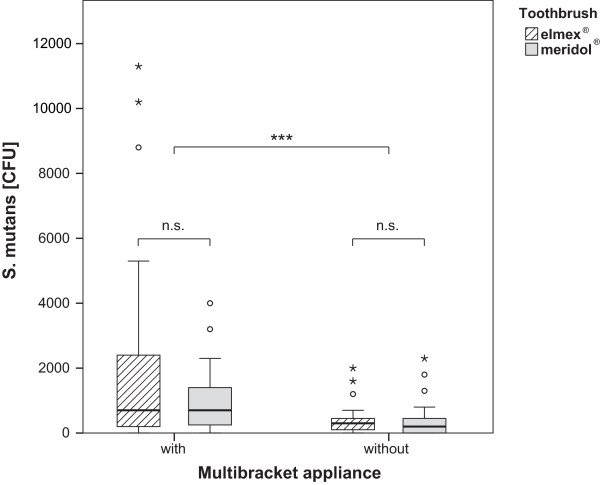
**Microbial colonisation with S. mutans in colony forming units (CFU) in patients with and without multibracket appliances using two types of toothbrushes.**

**Figure 3:**
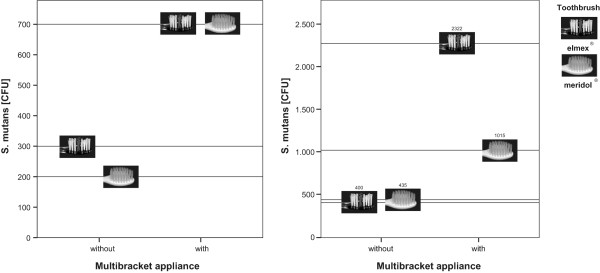
**Median (left) and mean numbers (right) of S. mutans in colony forming units (CFU) in patients with and without multibracket appliances using two types of toothbrushes.**

**Figure 4:**
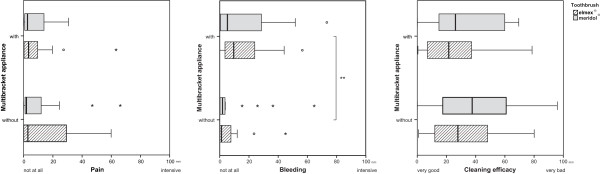
**Subjective perception of pain (left), gum bleeding (middle) and cleaning efficacy (right) in visual analog scale mm during toothbrushing with two different types of brushes.**

84% of the toothbrushes showed colonisation with *S. mutans*, but no difference was found between the two bristle designs (p = 0.6655). However, subjects with MB had significantly higher bacterial counts, independent of brush type (p = 0.0003) than subjects without MB (Figure 2). The highest percentage of contaminated brushes (96%) was found in group MBe, the lowest in group nMBm (70%) (Table 4). Regarding median numbers, subjects without MB harboured 200 (nMBm) and 300 (nMBe) CFU, whereas subjects with MB exhibited 700 CFU (MBm and MBe), respectively (Table 5 and Figure 3). Thus, multibracket appliances appear to enhance the retention of microorganisms on manual toothbrushes significantly. Regarding the mean values, the greatest difference was found between nMBe (400 CFU) and MBe (2322 CFU) (Figure 3). A growth of lactobacilli was observed on only 4 brushes and *Candida* could not be found on any brush. Hence no statistical analysis could be performed for these two microorganisms.Participants with fixed appliances reported an increased subjective bleeding tendency (p = 0.0065), which didn´t seem to be influenced by a certain toothbrush (p = 0.6018). The perception of pain could neither be related to orthodontic appliances (p = 0.6544) nor to a certain bristle design (p = 0.8639), as was the case when asking for the perceived cleaning efficacy (p = 0.3036 and p = 0.6975) (Figure 4) in all groups.

**Table 4:** **Number and percentage of colonised toothbrushes in the four experimental groups**

Microorganisms	S. mutans		Lactobacilli		Candida	
	n	%	n	%	n	%
MBe	22	96	4	17	0	0
MBm	18	90	0	0	0	0
nMBe	19	79	0	0	0	0
nMBm	14	70	0	0	0	0
**Total**	**73**	**84**	**4**	**5**	**0**	**0**

(MBe= MB and elmex® toothbrush, MBm= MB and meridol® toothbrush, nMBe= no MB and elmex® toothbrush, nMBm= no MB and meridol® toothbrush) and for the three microorganisms analysed.

**Table 5:** **Numbers of microorganisms in colony forming units (CFU) per brush head in the four experimental groups**

CFU per brush head	Minimum	Maximum	Median	Mean	SD
MBe	0	11300	700	2322	3400
MBm	0	4000	700	1015	1108
nMBe	0	2000	300	400	514
nMBm	0	2300	200	435	645

(MBe= MB and elmex® toothbrush, MBm= MB and meridol® toothbrush, nMBe= no MB and elmex® toothbrush, nMBm= no MB and meridol® toothbrush).

## Discussion

The aim of the present study was to evaluate the extent to which caries-associated microorganisms adhere to two manual toothbrushes differing in their bristle design and to analyze the impact of multibracket appliances on microbial retention. Despite the different background and age of the subjects, the groups seem comparable with regard to their oral hygiene habits. A higher consciousness for oral prophylaxis, as might be expected in dental students, could not be verified in previous studies [21]. On the other hand, one might also expect orthodontic patients to have a higher motivation for a good oral hygiene, implying a higher frequency of tooth brushing and thus more debris on the tooth brushes. Unfortunately, it is not possible to assure an identical brushing situation in all patients, resulting in a remaining degree of uncertainty as to whether or not oral hygiene habits were comparable between and within the two groups. As the inclusion criteria required a healthy or conservatively treated permanent dentition, it can be assumed that all participants had similar microbial conditions concerning caries-related germs and thus only differed in the presence of a MB appliance [22, 23]. The gender distribution was not homogenous, which doesn´t seem unusual bearing in mind that the request for orthodontic treatment, as well as for studying dentistry is much higher in females [24, 25]. As no gender-related difference in the microbial load of the brushes could be determined, a possible influence of this factor seems negligible.

Colonisation with *S. mutans* was found on 84% of the brushes, revealing the greatest variance between the groups MBe (96%) and nMBm (70%). The bristle design seemed to have no impact on the number of germs retained on the brush head. However, a significant difference became obvious when comparing brushes from subjects with MB to those without MB, indicating that brushes used by orthodontic patients with fixed appliances tend to harbor more microorganisms. A comparison of the present results to previous data in literature proved to be difficult due to the varying methods and study designs applied. Up to now, all microbial studies on toothbrushes were performed in vitro or in vivo on subjects without MB, so that only the amounts of microorganisms of the nMB groups are suitable for comparison. Only two authors refer to the number of *S. mutans* per brush head [23, 26]. After single use of new toothbrushes by patients with periodontal problems, Quirynen [26] isolated 2×10^6^ CFU, whereas the use of toothpaste reduced these numbers to 1,8x10^3^ CFU, which is in accordance to the numbers of *S. mutans* in our study.

A further explanation for the comparatively low numbers of *S. mutans* might be the reduction of germs achieved by drying during the intervals in which the brushes weren´t used. The extent to which the number of germs may be reduced varies: After 8 hours of air drying, Spolidorio et al. [27] weren´t able to detect any remaining *S. mutans*, whereas Svanberg [28] found the same bacteria even after 24 hours to the magnitude of 10^4^ CFU. To prevent reduction of germs due to drying after the brushes´ last use, sealable plastic bags were distributed, so brushes were gained under standardized conditions.

Wetzel et al. [19] compared different principles of filament anchoring and related the usual “multi-tufted” technique to an increased microbial retention, which is in accordance with Glass´ [29] statement that bacterial contamination may be decreased with less bristles per tuft and less tufts per brush head. Despite this, we didn´t find any microbial disadvantage of the meridol® toothbrush, which features an even more compact tufting than the elmex® brush. On the contrary, the elmex® toothbrush tended to be microbiologically inferior. Again this might be caused by the use of toothpaste, because brushes with many fine filaments, such as the meridol® toothbrush, not only harbor more bacteria but also more toothpaste [30] which maintains the antimicrobial effect among the bristles and perhaps leveled the differences between the two toothbrush-types in our investigation.

Retrospectively, it would have been helpful to have a baseline assessment of saliva samples. According to Jordan and LeBlanc [31] plaque and saliva samples correlate in terms of number and type of microorganisms. If a participant, for instance, provided low saliva counts of *S. mutans*, it would be hardly surprising if only few germs were found on his toothbrush. The present randomised study design, however, ensured a homogenous distribution of subjects with differing germ quantities and qualities, so a baseline-assessment did not seem indispensable.

Only 5% of the toothbrushes were colonized with lactobacilli, all of them belonged to group MBe, though the low percentage didn´t allow for statistical analysis. The rare incidence of these bacteria is surprising, regarding the fact that during MB-treatment the amount of lactobacilli are reported to increase considerably [32], which is mainly associated with the many retention sites a multibracket appliance offers [33]. Nies et al. [20] investigated the microbial contamination on 70 toothbrushes and found a consistent colonization with lactobacilli. Since we employed the same method, the frequent isolation of lactobacilli described by Nies et al. might be due to their subject materials, which were children with carious dentitions, whereas caries was an exclusion criterion in the present study.

*Candida* species were not found in any of our specimens, although an average oral prevalence of *C. albicans* is described to be between 25 to 75% [34]. About 20% of patients treated with MB become *Candida*-carriers which is probably caused by interaction between orthodontic appliance, virulence and host factors [3, 34]. Several authors report on isolating *C. albicans* from toothbrush heads [12, 20] and emphasize its good ability to colonize [35], whereas others also came upon very few, if any CFU [11]. As mentioned above, a baseline assessment might have been advantageous for explaining the absence of *C. albicans* on the brush heads in our investigation.

For assessing individual perceptions, such as brushing comfort, gum bleeding and cleaning efficacy of the brushes, a questionnaire with VAS was used, which is regarded superior to verbal methods when evaluating personal sensations [36]. By means of VAS there was no indication, that bristle design or the presence of MB affects the experience of pain during tooth brushing. Also the perceived cleaning efficacy was not correlated to one of these determinants. An observable bleeding during brushing, however, was related to fixed appliances, but not to the kind of brush used. The discussion in literature about what kind of toothbrush is best for use during orthodontic treatment is controversial. Electric toothbrushes are often compared with manual brushes, but generally are not regarded as superior, although they may provide a better approximal cleaning with reduction of the bleeding in this area [6, 7]. The enhanced bleeding tendency in patients with MB during tooth brushing coincides with the clinical appearance: although the salivary flow and intraoral pH increase, which both counteracts the development of caries, gingivitis is common and often hard to prevent.

Since the intraoral perception of a toothbrush depends on their design and bristle consistency [37], the meridol® brush is likely to convey a soft and pleasant feeling, although subjects described no difference concerning the cleaning efficacy of both brushes. Moreover conical and fine filaments are attributed to a better cleaning efficacy at the gingival margin and in approximal regions [8], which seems to be correlated to the flexibility of the filaments that may easily access the areas around the brackets and below the archwire. This should be assessed in future studies.

## Conclusion

Since toothbrushes of MB-patients, independent of their bristle design, had a higher microbial load than those of subjects without MB, a more frequent replacement of toothbrushes during MB treatment may be advisable.

Due to no differences between the two bristle designs, the recommendation for toothbrush-type should be made according to personal preferences of the patients, since it can be assumed that patients use a toothbrush more intensely when brushing with it is comfortable.

## References

[1] Glasl B, Ludwig B, Schopf P (2006). Prevalence and development of KIG-relevant symptoms in primary school students from Frankfurt am Main. J Orofac Orthop.

[2] Arslan SG, Akpolat N, Kama JD, Özer T, Hamamci O (2008). One-year follow-up of the effect of fixed orthodontic treatment on colonization by oral Candida. J Oral Pathol Med.

[3] Hägg U, Kaveewatcharanont P, Samaranayake YH, Samaranayake LP (2004). The effect of fixed orthodontic appliances on the oral carriage of Candida species and Enterobacteriaceae. Eur J Orthod.

[4] Petti S, Barbato E, Simonetti D´Arca A (1997). Effect of orthodontic therapy with fixed and removable appliances on oral microbiota: a six-month longitudinal study. New Microbiol.

[5] Faltermeier A, Bürgers R, Rosentritt M (2008). Bacterial adhesion of Streptococcus mutans to esthetic bracket materials. Am J Orthod Dentofacial Orthop.

[6] Thienpont V, Dermaut LR, van Maele G (2001). Comparative study of 2 electric and 2 manual toothbrushes in patients with fixed orthodontic appliances. Am J Orthod Dentofacial Orthop.

[7] Hickman J, Millett DT, Sander L, Brown E, Love J (2002). Powered vs manual tooth brushing in fixed appliance patients: a short term randomized clinical trial. Angle Orthod.

[8] Barnes CM, Covey DA, Shi X, Yankell SL (2009). Laboratory evaluations of a bi-level, extremely tapered bristled toothbrush and a conventional uniform bristled toothbrush. Am J Dent.

[9] Yankell SL, Shi X, Emling RC (2003). Laboratory evaluations of two toothbrushes for removal of artificial plaque above, around and below the gingival margin. J Clin Dent.

[10] Dörfer CE, von Bethlenfalvy ER, Kugel B, Pioch T (2003). Cleaning efficacy of a manual toothbrush with tapered filaments. Oral Health Prev Dent.

[11] Verran J, Leahy-Gilmartin AA (1996). Investigations into the microbial contamination of toothbrushes. Microbios.

[12] Noga K, Lange DE, Alai-Omid W (1976). Mykologische Untersuchungen an Zahnbürsten. Dtsch Zahnärztl Z.

[13] Glass RT, Jensen HG (1988). More on the contaminated toothbrush: the viral story. Quintessence Int.

[14] Aas JA, Griffen AL, Dardis SR, Lee AM, Olsen I, Dewhirst FE, Leys EJ, Paster BJ (2008). Bacteria of dental caries in primary and permanent teeth in children and young adults. J Clin Microbiol.

[15] Fitzgerald RJ, Keyes PH (1960). Demonstration of the etiologic role of streptococci in experimental caries in the hamster. J Am Dent Assoc.

[16] Badet C, Thebaud NB (2008). Ecology of lactobacilli in the oral cavity: a review of literature. Open Microbiol J.

[17] Hossain H, Ansari F, Schulz-Weidner N, Wetzel WE, Chakraborty T, Domann E (2003). Clonal identity of Candida albicans in the oral cavity and the gastrointestinal tract of pre-school children. Oral Microbiol Immunol.

[18] Pancherz H, Mühlich DP (1997). Entwicklung von Karies bei kieferorthopädischer Behandlung mit festsitzenden Apparaturen – Ein Vergleich von Zähnen mit und ohne Kariesvorschädigungen. Kieferorthop.

[19] Wetzel WE, Schaumburg C, Ansari F, Kröger T, Sziegoleit A (2005). Microbial contamination of toothbrushes with different principles of filament anchoring. J Am Dent Assoc.

[20] Nies SM, Kröger T, Ansari F, Schaumburg C, Wetzel WE (2008). Keimbesiedlung an Zahnbürsten mit unterschiedlichen Borstenbündelbesteckungen. Oralprohylaxe Kinderzahnheilkd.

[21] Lang NP, Cumming BR, Löe HA (1977). Oral hygiene and gingival health in Danish dental students and faculty. Community Dent Oral Epidemiol.

[22] Percival RS, Challacombe SJ, Marsh PD (1991). Age-related microbiological changes in the salivary and plaque microflora of healthy adults. J Med Microbiol.

[23] Kozai K, Iwai T, Miura K (1989). Residual contamination of toothbrushes by microorganisms. ASDC J Dent Child.

[24] Krey KF, Hirsch C (2012). Frequency of orthodontic treatment in German children and adolescents: influence of age, gender, and socio-economic status. Eur J Orthod.

[25] Wheeler TT, McGorray SP, Yurkiewicz L, Keeling SD, King GJ (1994). Orthodontic treatment demand and need in third and fourth grade schoolchildren. Am J Orthod Dentofacial Orthop.

[26] Quirynen M, De Soete M, Pauwels M, Goossens K, Teughels W, van Eldere J, van Steenberghe D (2001). Bacterial survival rate on tooth- and interdental brushes in relation to the use of toothpaste. J Clin Periodontol.

[27] Spolidorio DM, Goto E, Negrini Tde C, Spolidorio LC (2003). Viability of Streptococcus mutans on transparent and opaque toothbrushes. J Dent Hyg.

[28] Svanberg M (1978). Contamination of toothpaste and toothbrush by Streptococcus mutans. Scand J Dent Res.

[29] Glass RT (1992). Toothbrush types and retention of microorganisms: how to choose a biologically sound toothbrush. J Okla Dent Assoc.

[30] Dyer D, Addy M, Newcombe RG (2000). Studies in vitro of abrasion by different manual toothbrush heads and a standard toothpaste. J Clin Periodontol.

[31] Jordan C, LeBlanc DJ (2002). Influences of orthodontic appliances on oral populations of mutans streptococci. Oral Microbiol Immunol.

[32] Peros K, Mestrovic S, Anic-Milosevic S, Slaj M (2011). Salivary microbial and nonmicrobial parameters in children with fixed orthodontic appliances. Angle Orthod.

[33] Kupietzky A, Majumdar AK, Shey Z, Binder R, Matheson PB (2005). Colony forming unit levels of salivary Lactobacilli and Streptococcus mutans in orthodontic patients. J Clin Pediatr Dent.

[34] Hibino K, Wong RW, Hägg U, Samaranayake LP (2009). The effects of orthodontic appliances on Candida in the human mouth. Int J Paediatr Dent.

[35] Bunetel L, Tricot-Doleux S, Agnani G, Bonnaure-Mallet M (2000). In vitro evaluation of the retention of three species of pathogenic microorganisms by three different types of toothbrush. Oral Microbiol Immunol.

[36] Huskisson EC (1974). Measurement of pain. Lancet.

[37] Golding PS (1982). The development of the toothbrush. Part 2. The modern toothbrush. Dent Health.

